# Risk-Stratified Approach to Breast Cancer Screening in Canada: Women’s Knowledge of the Legislative Context and Concerns about Discrimination from Genetic and Other Predictive Health Data

**DOI:** 10.3390/jpm11080726

**Published:** 2021-07-27

**Authors:** Samuel Alarie, Julie Hagan, Gratien Dalpé, Sina Faraji, Cynthia Mbuya-Bienge, Hermann Nabi, Nora Pashayan, Jennifer D. Brooks, Michel Dorval, Jocelyne Chiquette, Laurence Eloy, Annie Turgeon, Laurence Lambert-Côté, Jean-Sébastien Paquette, Meghan J. Walker, Julie Lapointe, Palmira Granados Moreno, Kristina Blackmore, Michael Wolfson, Mireille Broeders, The PERSPECTIVE I&I Study Group, Bartha M. Knoppers, Anna M. Chiarelli, Jacques Simard, Yann Joly

**Affiliations:** 1 Centre of Genomics and Policy, McGill University, Montreal, QC H3A 0G1, Canada; samuel.alarie@umontreal.ca (S.A.); gratien.dalpe@mcgill.ca (G.D.); sina.faraji@mcgill.ca (S.F.); palmira.granadosmoreno@mcgill.ca (P.G.M.); bartha.knoppers@mcgill.ca (B.M.K.); yann.joly@mcgill.ca (Y.J.); 2 CHU de Québec-Université Laval Research Center, Quebec City, QC G1V 4G2, Canada; cynthia.mbuya-bienge.1@ulaval.ca (C.M.-B.); Hermann.Nabi@fmed.ulaval.ca (H.N.); Michel.Dorval@crchudequebec.ulaval.ca (M.D.); jocelyne.chiquette@crchudequebec.ulaval.ca (J.C.); Annie.Turgeon@crchudequebec.ulaval.ca (A.T.); Laurence.Lambert-Cote@crchudequebec.ulaval.ca (L.L.-C.); Julie.Lapointe@crchudequebec.ulaval.ca (J.L.); jacques.simard@crchudequebec.ulaval.ca (J.S.); 3 Department of Social and Preventive Medicine, Faculty of Medicine, Université Laval, Quebec City, QC G1V 0A6, Canada; 4 Université Laval Cancer Research Center, Quebec City, QC G1R 3S3, Canada; 5 Department of Applied Health Research, Institute of Epidemiology and Healthcare, University College London, London WC1E 6BT, UK; n.pashayan@ucl.ac.uk; 6 Dalla Lana School of Public Health, University of Toronto, Toronto, ON M5S 1A1, Canada; jennifer.brooks@utoronto.ca (J.D.B.); Meghan.Walker@ontariohealth.ca (M.J.W.); Anna.Chiarelli@ontariohealth.ca (A.M.C.); 7 Faculty of Pharmacy, Université Laval, Quebec City, QC G1V 4G2, Canada; 8 CISSS de Chaudière-Appalaches Research Center, Lévis, QC G6V 3Z1, Canada; 9 CHU de Québec-Université Laval, Quebec City, QC G1S 4L8, Canada; 10 Département de Médecine Familiale et de Médecine D’urgence, Université Laval, Quebec City, QC G1V 4G2, Canada; jspaquette.lab@gmail.com; 11 Québec Cancer Program, Ministère de la Santé et des Services Sociaux, Quebec City, QC G1S 2M1, Canada; laurence.eloy@msss.gouv.qc.ca; 12 Department of Social and Preventive Medicine, CISSS de Lanaudière-Université Laval, Quebec City, QC G1V 0A6, Canada; 13 Ontario Health (Cancer Care Ontario), Toronto, ON M5G 2L3, Canada; kristina.blackmore@ontariohealth.ca; 14 School of Epidemiology and Public Health, University of Ottawa, Ottawa, ON K1G 5Z3, Canada; Michael.Wolfson@uOttawa.ca; 15 Radboud Institute for Health Sciences, Radboud University Medical Center, 525 EZ Nijmegen, The Netherlands; Mireille.Broeders@radboudumc.nl; 16 Dutch Expert Centre for Screening, 6538 SW Nijmegen, The Netherlands; 17 Department of Molecular Medicine, Faculty of Medicine, Université Laval, Quebec City, QC G1V 4G2, Canada

**Keywords:** breast cancer, risk-stratified screening, population survey, genetic discrimination, women’s perspectives

## Abstract

The success of risk-stratified approaches in improving population-based breast cancer screening programs depends in no small part on women’s buy-in. Fear of genetic discrimination (GD) could be a potential barrier to genetic testing uptake as part of risk assessment. Thus, the objective of this study was twofold. First, to evaluate Canadian women’s knowledge of the legislative context governing GD. Second, to assess their concerns about the possible use of breast cancer risk levels by insurance companies or employers. We use a cross-sectional survey of 4293 (age: 30–69) women, conducted in four Canadian provinces (Alberta, British Colombia, Ontario and Québec). Canadian women’s knowledge of the regulatory framework for GD is relatively limited, with some gaps and misconceptions noted. About a third (34.7%) of the participants had a lot of concerns about the use of their health information by employers or insurers; another third had some concerns (31.9%), while 20% had no concerns. There is a need to further educate and inform the Canadian public about GD and the legal protections that exist to prevent it. Enhanced knowledge could facilitate the implementation and uptake of risk prediction informed by genetic factors, such as the risk-stratified approach to breast cancer screening that includes risk levels.

## 1. Introduction

New approaches for population-wide breast cancer screening, incorporating polygenic risk score (PRS) in risk assessment models, are in development. These approaches could improve current age-based breast cancer screening programs [1,2,3,4,5]. While promising, the implementation of such risk-stratified approaches depends on several factors, including the uptake by women of a genetic test that allows, in combination with other personal and lifestyle factors, to estimate their breast cancer risk level. Several authors have identified the fear of genetic discrimination (GD) as one of the barriers to genetic testing for breast cancer screening [6,7,8,9,10]. Most existing studies addressing GD concerns as a possible barrier to breast cancer screening focus on high-penetrance rare *BRCA1* and *BRCA2* mutations [6,7,8,10], rather than low-penetrance common variants (SNPs) included in a polygenic risk score (PRS) [11] and other such predictive health data. Although the personalized risk level of the risk-stratified approach is distinct from a genetic test result for mutations in rare predisposition genes (e.g., *BRCA1*, *BRCA2*), women may nevertheless have concerns about genetic testing for PRS. As Lewis and Green recently pointed out, “we might expect many individuals to forgo the potential upsides of receiving PRS because of fear that the information will be used against them in insurance pricing or availability” [12] (p. 5). It is therefore pressing to assess women’s perceptions of the use of their genetic and health data in the context of a risk assessment that includes PRS.

GD generally refers to treating an individual or group unfairly or prejudicially based on their genetic characteristics [13]. While some earlier publications suggested that concerns about GD may be more common than actual occurrences [14,15], many authors have since argued that GD does occur, but is often underreported [16,17,18]. Laws to prevent GD have been adopted in various countries (e.g., *Genetic Non-Discrimination Act*, Canada, *Bundesrat Gesetz über genetische Untersuchungen bei Menschen*, Germany, and *Genetic Information Non-discrimination Act*, United States) [13]. According to the literature, general awareness of policies surrounding GD is low [19,20,21]. In addition, other factors (e.g., personal and family history, past experience of discrimination) may influence perceptions of GD [21,22,23,24]. To our knowledge, no quantitative study on attitudes towards and knowledge of the legislative context surrounding discrimination from genetic and other predictive health data has been conducted with a large sample of participants in Canada.

Considering the above, we conducted a study to better understand the extent of concerns about discrimination from genetic and other predictive health data in relation to the implementation of a risk-based approach to breast cancer screening. Our research objectives are twofold. First, is to evaluate Canadian women’s knowledge of specific issues related to different aspects of the legislative context governing GD. Second, is to assess their concerns about the possible use of breast cancer risk levels by insurance companies or employers.

## 2. Materials and Methods

This study is part of the Canadian PERSPECTIVE I&I (Personalized risk assessment for prevention and early detection of breast cancer: Integration and Implementation) project, which is one of the current initiatives exploring the potential of risk stratification to improve population-based breast cancer screening [4]. We used an online cross-sectional survey of 4293 women (age: 30–69) from an online panel that was conducted in four provinces in Canada (Alberta, British Colombia, Ontario and Quebec) and took place between 19 and 26 February 2019.

The questionnaire was developed and translated in both English and French by the research team [25]. Details of the questionnaire design were published previously [25]. The development of the questionnaire was based on previous surveys on the same topic. Questions specifically related to GD were developed based on the expertise of the team members in this area (G.D., Y.J.). The research team included social scientists, epidemiologists and clinicians. The questionnaire was pilot tested for clarity and consistency (*n* = 100).

### 2.1. Measures

#### 2.1.1. Sociodemographic Variables

Sociodemographic variables included participants’ self-reported age, country of birth, marital status, educational level, employment status, annual family income and province of residence. Participants were also asked about the status and/or potential interest in getting private health/life insurance: “With regard to life insurance and private (non-governmental) health insurance, please select which statement best describes your situation: (a) I currently have life insurance and/or private health insurance; (b) I do not currently have life insurance and/or private health insurance, but I am or could become interested in getting one or both in the future; (c) I am not interested in getting life insurance and/or private health insurance; (d) Prefer not to answer”. Participants were also asked about their personal history of genetic testing or that of a blood-relative in two questions: “(1) Have you ever had a genetic test for breast cancer (e.g., a blood test that checks the features of the BRCA genes)?” and “(2) Have any of your blood relatives ever had a genetic test for breast cancer (e.g., a blood test that checks the features of the BRCA genes)?” Participants could answer “Yes”/”No”/”Don’t know”/”Prefer not to answer”.

#### 2.1.2. Outcome Variables

The level of knowledge about discrimination based on genetic and other predictive health data and about the legal framework for potentially objectionable practices was measured by 9 “True”/”False”/”I don’t know” statements as shown in Table 1.

Level of concern was measured by one question: “Please indicate which statement best describes your state of mind about the potential use of information about your breast cancer risk level by employers or insurance companies. Participants were asked to select a multiple-choice answer: (a) I have no concerns about how my breast cancer risk level might be used by employers or insurance companies; (b) I have some concerns about how my breast cancer risk level might be used by employers or insurance companies; (c) I have a lot of concerns about how my breast cancer risk level might be used by employers or insurance companies; (d) Don’t know; (e) Prefer not to answer”.

## 3. Results

### 3.1. Sample Characteristics

We performed a chi-square analysis crossing both concern and knowledge levels with sociodemographic variables [26]. To improve data quality, we excluded 324 participants from the original sample of 4293, due to them answering “I don’t know” to all 9 knowledge questions, which we identified as straight lining behavior [27]. The removal of straight liners left us with a total of 3969 participants. Table 2 presents the sociodemographic characteristics of this final sample.

### 3.2. Knowledge

In line with the existing literature [7,19,20,21], our results show that Canadian women’s knowledge of the regulatory framework for GD is relatively limited. Women’s lack of knowledge about the legislative framework for genetic discrimination has two dimensions: (1) absence of knowledge, represented by the women who chose to answer “Don’t know” to specific statements, and (2) misconception, represented by the rates of incorrect answers for any given specific statements.

Among the participants, the average rate of right answer was 50.94% and goes up to 82.55% for Q6, on the need for employers to have employees’ consent to access information in their health records, and down to 16.97% for Q4, on the potential importance of the level of breast cancer risk to employers. The average rate of wrong answers is 25.43%, which varies from 5.16% for Q6 to 59.61% for Q4. A personal history of genetic test is associated with slightly better knowledge for Q4 (*p* = 0.012) and Q9 (*p* = 0.016), while having a blood relative with a history of genetic test is associated with slightly worse knowledge for Q1 (*p* = 0.050) and Q8 (*p* = 0.001).

The “Don’t know” rate has an average of 24.02% (from 12.29% to 31.15%). As shown in Table 3, only 12.29% of respondents answered “Don’t know” to Q6. The other three statements with higher than average “Don’t know” rates were Q2, with 19.63%, Q5, with 21.65%, and Q4, with 23.42%. Q6, Q2 and Q5 are also the statements for which the rates of correct answers are among the highest with, respectively, 82.55%, 62.78% and 66.96%. On the other hand, Q4 has both a high “Don’t know” rate (23.42%) and the lowest correct answer rate, with 16.97%. Such results show a moderately high level of understanding about insurance companies’ access to health records. There is a lower understanding of and higher misconceptions about employers’ interests in employees’ health information.

As shown in Table 4, sociodemographic factors that had a significant effect on responses to Q1, assessing knowledge about the potential impact of the level of breast cancer risk on a woman’s ability to purchase insurance, were province of residence (*p* = 0.019 *), insurance status (*p*= 0.037 *) and education (*p* = 0.018 *). Respondents who indicated that they were currently uninsured but were interested in obtaining insurance products in the future had a significantly lower correct response rate (56.75%) than those who indicated that they were uninsured and not interested in obtaining insurance products, which had a correct response rate of 65.96%. Women with a university degree had a higher rate of correct response (64.09%) than women with only a high school diploma or less (57.06%).

Only insurance status (*p* = 0.001 **) and family income (*p* = 0.006 **) had a significant effect on responses to Q2, assessing knowledge about the potential impact of the level of breast cancer risk on a woman’s ability to get a job. Those who are insured have a higher correct response rate (79.96%) than those who are not insured but would like to be (73.24%). Moreover, those with incomes over CAD 80k have a higher correct response rate (80.75%) than those with incomes in the less than CAD 20k and CAD 20–40k brackets, whose correct response rate was 75.21% and 72.53%, respectively. Although the percentages are small, we note that the rate of correct responses increases as the income bracket increases.

Province of residence (*p* < 0.001 **), education (*p* = 0.008 **) and employment status (*p* = 0.014 *) had a significant effect on respondents’ answers to Q3 about the potential importance of the level of breast cancer risk to insurers. Indeed, Quebec has a lower rate of correct answers (29.42%) than BC (53.46%), AB (53.06%) and Ontario (51.32%). Respondents with a university degree have a higher rate of correct answers (50.75%) than the other two categories, post-secondary education (45.16%) and high school or less (43.56%). Those who are currently employed have a higher rate of correct answers (48.19%) than the retired, who have a correct answer rate of 41.30%.

Only province of residence (*p* = 0.026 *) had a significant effect on the responses to Q4 about the potential importance of the level of breast cancer risk to employers. It is important to note that Q4 is also the response with the lowest rate of correct answers. The province of Quebec has the lowest rate of correct answers (18.44%), although this rate is also relatively low for the other provinces surveyed. Alberta respondents had a 22.79% correct response rate, while British Columbia and Ontario had 23.77% and 24.03% correct response rate, respectively, for this question.

Age (*p* = 0.001 **), insurance status (*p* = 0.008 **), employment status (*p* = 0.001 **) and family income (*p* = 0.004 **) had a significant effect on responses to Q5, appraising knowledge about insurers accessing information (including the level of breast cancer risk) in women’s health records without their approval. The 60–69 age category has a significantly higher rate of correct response (89.32%) than the respondents in the 30–39 age category (84.41%), the 40–49 age category (82.33%) and the 50–59 age category (85.48%). Those who report having insurance have a higher rate of correct responses (86.72%) than those who do not currently have insurance but are interested in having insurance in the future (83.81%) and those who do not have insurance and are not interested in purchasing insurance coverage in the future (80.91%). People who do not work have a lower rate of correct answers (81.01%) than workers (85.72%) and retirees (83.34%). Finally, respondents with an income under CAD 20k have a lower rate of correct answers 79.92%) than any other income category. Again, we note that the rate of correct answers increases as the income bracket increases.

Age (*p* = 0.014 *), province of residence (*p* = 0.003 **), insurance status (*p* = 0.020 *), marital status (*p* = 0.007 **) and employment status (*p* = 0.007 **) all had significant effects on responses to Q6, concerning knowledge about employers accessing information in women’s health records without their approval. When it comes to knowledge about employers’ access to health information, respondents aged 60–69 had a higher rate of correct answers (95.59%) than any other category. Although the differences are small, we notice that the rate of correct answers increases with the age of the respondents, with the 30–39 years old having the lowest rate of correct answers (92.00%). Again, Quebecers have a lower rate of correct response (91.82%) than Alberta (95.80%), British Columbia (94.05%) and Ontario (94.82%). The trend is also confirmed regarding insurance status, with respondents who have insurance showing a higher rate of correct responses (94.94%) than those without insurance and not interested in obtaining it (91.88%). Retirees had a higher rate of correct answers (96.26%) than workers (93.99%) and those who do not work (92.30%).

Only country of birth had a significant effect (*p* = 0.003 **) on answers to Q7, evaluating knowledge about the possibility that a woman who does not give access to her health record could be refused life/health insurance. Respondents born outside of Canada had a lower rate of correct answers (73.97%) than those born in Canada (80.32%).

Province of residence (*p* = 0.037 *) and country of birth (*p* < 0.001 **) had significant effects on the responses to Q8, assessing knowledge about the likelihood that an insurance contract may be cancelled if important health information (including the level of breast cancer risk) is not disclosed to insurers when purchasing or renewing a policy. Differences between regions are small, but again Quebec has a lower rate of correct responses (81.79%) than the other provinces, which have correct response rates ranging from 84.91%, for Alberta, to 85.64%, for Ontario, and 86.92%, for British Columbia, which has the highest correct response rate. Similar to what we observed for Q7, respondents born outside of Canada have a lower rate of correct responses (78.92%) than respondents born in Canada (85.78%). Sociodemographic information did not have any significant effect on Q9.

### 3.3. Concerns

Respondents’ concerns about discrimination based on genetic and health data are divided between those who are very concerned, those who have some concerns and those who are not concerned at all about the possible misuse of their personal data by employers or insurers. In terms of frequencies, about one third (34.7%) of the participants had a lot of concerns over the possible misuse of their personal data by employers or insurers. Another third had some concerns (31.9%), while 20% had no concerns. One in eight (12.2%) did not know if they are concerned about such a topic and 1.2% preferred not to answer.

As shown in Table 5, among the sociodemographic variables, age (*p* < 0.001), province of residence (*p* < 0.001), education level (*p* < 0.001), employment status (*p* < 0.001) and family income (*p* = 0.004) had a significant chi-square when crossed with the level of concerns about how their breast cancer risk level might be used by employers or insurance companies, excluding the “Don’t know” and “Prefer not to answer” categories. The country of birth (*p* = 0.586) and the marital status (*p* = 0.789) were not significant when crossed with the concern level. The status of insurance had a significant chi-square (*p* < 0.001), while the history of genetic test (*p* = 0.755) and the blood-relative history of genetic test (*p* = 0.218) were not significant.

It is important to note that, with respect to concerns about the potential use of their breast cancer risk level by insurers or employers, age has an effect for those who report having “some concerns” and “a lot of concerns”, but not for those who have no concerns. A greater percentage of Quebec women are in the “no concerns” category (31%), compared to respondents from other provinces. While slightly less than a third of Quebec women claim to have “no concerns”, the percentage of women who chose that answer is around one in five in British Columbia (20%), Alberta (20%) and Ontario (22%). With respect to the effect of education on respondents’ level of concerns, there were significant differences in all categories. The percentage of women with a high school diploma or less saying they were not concerned was higher (30%) than for those with post-secondary education (25%), which is higher than for those with a university degree (16%). The percentage of women with a university degree who selected the response “Some concerns” was higher than for those with a high school diploma or less (35%) or post-secondary education (34%) who selected the same response. More women with a university education reported being very concerned than those with a high school diploma (35%). With respect to employment status, there are significant differences in the “no concerns” and “some concerns” categories, but not for the answer “lot of concerns”. Women who do not work (26%) and retirees (28%) were more likely to have chosen the “no concerns” response than those who work (21%). Retired women were less likely to say they have “some concerns” (30%) compared to those who do not work (36%) and those who do (40%). Regarding income, there were significant differences in the “no concerns” category, with those earning CAD 20k and under being more likely to choose “no concerns” (32%) than women earning CAD 20k–40k (28%), those with an income CAD 40k–60k (22%) and those having an income of CAD 80k and over (22%).

## 4. Discussion

Our objective was to better understand concerns about discrimination based on genetic and other predictive health data in relation to the implementation of a risk-based approach to breast cancer screening. To this end, we first assessed Canadian women’s knowledge of the legislative context governing GD. Second, we sought to assess Canadian women’s concerns about the possible use of breast cancer risk levels by insurance companies or employers.

### 4.1. Knowledge

Knowledge of the legal framework governing the use of personal genetic data by employers and insurers is a subject that is quite hard to master for laypeople [28]. Thus, it is not surprising that knowledge and awareness of such regulation are relatively low in Canada, as this was also noted by several empirical studies conducted primarily in the United States [7,19,20,21]. In Canada, a previous breast cancer pilot study also showed that most participants were not aware of a duty to disclose genetic test results when applying for personal insurance [9]. Our results provide further information on variables that can influence the level of knowledge on GD and show that, while some aspects (e.g., insurance companies’ access to health records) are relatively well understood, others (e.g., employers’ interests in employees’ health information) still lack clarity.

Cross-tabulations of sociodemographic and outcome variables show that some, but not all, sociodemographic characteristics have an effect on outcomes. For example, province of residence, education, insurance status and employment status had an effect on knowledge about the possible impact of the level of breast cancer risk on insurability and employability, as well as knowledge about employers’ and insurers’ access to health information. There is a correlation between educational attainment and province of residence (p < 0.001), which may explain, in part, the differences between provinces. For example, Quebec has the lowest proportion of university graduates (225/973 = 23.1%) compared to Ontario (370/1002 = 36.9%), Alberta (278/1002 = 27.7%) and British Columbia (285/989 = 28.8%). This provides avenues for reflection regarding communication strategies to be adopted in the context of the implementation of a risk-based approach to breast cancer screening. For example, in the Canadian context, approaches tailoring communication strategies to specific province may be good practice.

Personal experience with genetic testing for cancer is associated with greater awareness of some employability issues. We note that having a blood relative with a history of genetic testing is associated with misconceptions about insurance (e.g., about the potential impact of breast cancer risk level on a woman’s ability to purchase insurance and the possibility that an insurance contract will be voided if important health information, including breast cancer risk level) is not disclosed to insurers when purchasing or renewing a policy. This underscores the need to properly inform individuals about the practices of insurance companies and the existing regulatory framework.

The mixed results regarding respondents’ knowledge highlight that there will be a need for healthcare providers and genetic counsellors to communicate relevant information to women undergoing risk-stratified screening. In the context of genetic testing, it has been shown that women were “more likely to pursue testing if their physicians had informed them […] that there are legal protections against GD” [4]. Our results also point to the possibility that some segments of the population (e.g., those who do not have insurance, newcomers, etc.) may have specific informational needs with respect to the legal framework governing GD in Canada.

Genetic counsellors are generally consulted for discussing the risk and benefit of taking a genetic test with patients and could play a key role, along with other healthcare providers, in conveying information about insurability and employment impact of genetic testing to asymptomatic women undergoing risk-stratified breast cancer screening. However, genetic counsellors have expressed their limited knowledge of legal protections and policies concerning insurers’ practices in the context of genetic test results [29]. Furthermore, in a population-based approach, genetic counselors are also unlikely to be involved with large numbers of women, although it can be assumed that they could be involved with women at high risk. Decision-makers, particularly public health authorities, will have a role to play in keeping up to date with the regulations and providing accurate information to healthcare providers and genetic counsellors about the potential impact of such regulations on proposed screening programs. This is in line with recent work by Joly, Dalpé and Pinkesz, which recommends the implementation of “dynamic and nuanced information campaigns on GD and the ideal methods to prevent it” [30].

### 4.2. Concerns

Our results were relatively balanced with about one-third of participants having a great deal of concern about employers or insurers using their information, another third having some concern and the final third responding that they either have no concern or do not know. This is similar to the results of a survey of the general population in four American states, where over two-thirds of respondents were at least somewhat concerned about life insurance companies using their genetic test results to determine life insurance coverage and costs [20]. Despite the adoption of the *Genetic Non-Discrimination Act* in Canada in May 2017—creating criminal prohibitions against imposing genetic testing or forcing the disclosure of genetic test results in contractual agreements or the provision of goods and services—our results seem to indicate both that there has been little evolution in perceptions and that the Canadian public has a similar level of concern as the American public did 10 years ago. However, the adoption of the *Genetic Non-Discrimination Act* took place with very limited public discussion, which may account for the unchanged level of concern.

Respondents from the province of Quebec reported lower levels of concern about the potential use of breast cancer risk level information. Differential exposure to news about GD and the legislative framework that governs it could explain this specificity. There also seem to be a difference between the lower brackets of income when compared to the higher brackets. Both higher education and higher income levels appear to increase the level of concern. Interestingly, there were greater levels of concern among women aged 50–69, when compared to women aged 30–49. The fact that older women appear to be more concerned about discrimination may have an effect on their uptake of a program based on risk stratification. One suggestion would be to devote more resources to counseling this age group.

## 5. Conclusions

As with any study, this study has some limitations. First, it should be noted that this empirical study is exploratory and descriptive in nature and that subsequent studies, including multivariate analyses, can be conducted to further analyze and confirm the observed associations. Subsequent hypothesis-driven analysis could also be conducted to assess possible correlations between the level of concern and knowledge of specific GD regulatory issues in Canada. Adding the possibility to answer “I don’t know” to knowledge-based questions adds a level of difficulty to the analysis of data. However, the high number of participants who answered “I don’t know” to all nine knowledge-based questions tells us that a large percentage of the population is not willing to commit to an answer, which, in itself, is informative about the level of knowledge of the lay public in the legislative context governing discrimination from genetic and other predictive health data.

The constitutionality of the GNDA being debated in courts at the time of the survey—the Court of Appeal of Quebec had invalidated some provisions of the Act [31]—may also have created legal uncertainty and influenced the responses of some of the respondents. While both are limitations, they do support our conclusion that there is a need to further educate and inform the Canadian public about GD and to provide up-to-date information on the legal protections that exist to prevent it. It is important to keep in mind that the high rate of incorrect responses, as with Q4, reflects the complexity, nuances and sometimes ambiguity of the issues related to the evolving legal framework for GD. This is something that will need to be considered for the future implementation of screening approaches that include risk level categories informed by genetic factors, such as the risk stratification approach to breast cancer screening. Better knowledge of the existing protections against GD could facilitate the implementation and uptake of these promising approaches for women’s health in Canada.

## Figures and Tables

**Table 1:** Statements used to assess knowledge.

Statements	Correct Answer
Q1. The level of breast cancer risk has no impact on a woman’s ability to purchase insurance	False
Q2. The level of breast cancer risk has no impact on a woman’s ability to get a job	False
Q3. Unless the level of breast cancer risk requires medical attention or monitoring, it will not be important to an insurer	False
Q4. Unless the level of breast cancer risk requires medical attention or monitoring, it will not be important to an employer	False
Q5. Insurers cannot access any information (including the level of breast cancer risk) in women’s health records without their approval	True
Q6. Employers cannot access any information (including the level of breast cancer risk) in women’s health records without their approval	True
Q7. A woman who does not give access to her health record could be refused life / health insurance	True
Q8. An insurance contract might be cancelled if important health information (including level of breast cancer risk) is not disclosed to insurers when purchasing or renewing a policy	True
Q9. In some circumstances, a woman applying for a job who does not give approval to access her health record might lose the job opportunity	True

**Table 2:** Sociodemographic characteristics.

Characteristics	Participants (N = 3969)
**Age**	
30–39	972 (24.5%)
40–49	975 (24.6%)
50–59	1017 (25.6%)
60–69	1005 (25.3%)
**Province**	
Alberta	1002 (25.2%)
British Columbia	989 (24.9%)
Ontario	1003 (25.3%)
Quebec	975 (24.6%)
**Country of birth**	
Canada	3344 (84.3%)
Others	605 (15.2%)
**Education**	
High school or less	1058 (26.7%)
Post-secondary	1750 (44.1%)
University degree	1158 (29.2%)
**Marital status**	
Married or with partner	2475 (62.4%)
Divorced, separated, or widowed	725 (18.3%)
Single or never married	726 (18.3%)
**Employment status**	
Working	2355 (59.3%)
Not working	789 (19.9%)
Retired	784 (19.8%)
**Family income**	
Less than CAD 20,000	298 (7.5%)
CAD 20,000–39,999	581 (14.6%)
CAD 40,000–59,999	692 (17.4%)
CAD 60,000–79,999	553 (13.9%)
More than CAD 79,999	1337 (33.7%)

**Table 3:** Proportions of correct/incorrect/“Don’t know” answers by statement.

Statements	Correct Answers	Incorrect Answers	“Don’t Know”
Q1	1645 (41.56%)	1080 (27.29%)	1233 (31.15%)
Q2	2488 (62.78%)	697 (17.59%)	778 (19.63%)
Q3	1349 (34.06%)	1557 (39.31%)	1055 (26.63%)
Q4	672 (16.97%)	2360 (59.61%)	927 (23.42%)
Q5	2651 (66.96%)	451 (11.39%)	857 (21.65%)
Q6	3265 (82.55%)	204 (5.16%)	486 (12.29%)
Q7	2289 (57.82%)	597 (15.08%)	1073 (27.1%)
Q8	2497 (63.06%)	450 (11.36%)	1013 (25.58%)
Q9	1153 (29.12%)	1667 (42.11%)	1139 (28.77%)

**Table 5:** Correlations of sociodemographic characteristics and concern.

Characteristics	Chi-Square *p* Value
Age	*p* < 0.001
Province	*p* < 0.001
Country of birth	*p* = 0.586
Education	*p* < 0.001
Marital status	*p* = 0.789
Employment status	*p* < 0.001
Family income	*p* = 0.004
Insurance status	*p* < 0.001
Personal test	*p* = 0.755
Family test	*p* = 0.218

**Table 4:** Correlations of sociodemographic characteristics and answers to knowledge statements ^†^.

**Characteristics**	**Q1**	**Q2**	**Q3**	**Q4**	**Q5**
**Age**	*p* = 0.682	*p* = 0.581	*p* = 0.508	*p* = 0.070	*p* = 0.001 **
	**Correct answers**	**Correct answers**	**Correct answers**	**Correct answers**	**Correct answers**
30–39 40–49 50–59 60–69	419/692 (60.55%) 412/669 (61.58%) 433/712 (60.81%) 381/652 (58.44%)	595/772 (77.07%) 605/785 (77.07%) 647/819 (79.00%) 641/809 (79.23%)	346/715 (48.39%) 329/697 (47.20%) 337/749 (44.99%) 337/745 (45.23%)	159/743 (21.40%) 181/749 (24.17%) 182/762 (23.88%) 150/778 (19.28%)	628/744 (84.41%) 615/747 (82.33%) 689/806 (85.48%) 719/805 (89.32%)
**Province**	*p* = 0.019 *	*p* = 0.975	*p* < 0.001 **	*p* = 0.026 *	*p* = 0.424
	**Correct answers**	**Correct answers**	**Correct answers**	**Correct answers**	**Correct answers**
Alberta British Columbia Ontario Quebec	437/698 (62.61%) 404/640 (63.13%) 413/685 (60.29%) 391/702 (55.70%)	633/805 (78.63%) 595/763 (77.98%) 631/812 (77.71%) 629/805 (78.14%)	390/735 (53.06%) 363/679 (53.46%) 368/717 (51.32%) 228/775 (29.42%)	175/768 (22.79%) 169/711 (23.77%) 179/745 (24.03%) 149/808 (18.44%)	678/778 (87.15%) 628/739 (84.98%) 652/763 (85.45%) 693/822 (84.31%)
**Country of birth**	*p* = 0.478	*p* = 0.449	*p* = 0.374	*p* = 0.302	*p* = 0.421
	**Correct answers**	**Correct answers**	**Correct answers**	**Correct answers**	**Correct answers**
Canada Others	1394/2293 (60.79%) 247/419 (58.95%)	2103/2683 (78.38%) 375/488 (76.84%)	1134/2454 (46.21%) 212/437 (48.51%)	566/2583 (21.91%) 105/435 (24.14%)	2243/2618 (85.68%) 396/470 (84.26%)
**Education**	*p* = 0.018 *	*p* = 0.278	*p* = 0.008 **	*p* = 0.568	*p* = 0.362
	**Correct answers**	**Correct answers**	**Correct answers**	**Correct answers**	**Correct answers**
High school or less Post-secondary University degree	408/715 (57.06%) 722/1206 (59.87%) 514/802 (64.09%)	649/839 (77.35%) 1142/1438 (79.42%) 697/907 (76.85%)	318/730 (43.56%) 592/1311 (45.16%) 438/863 (50.75%)	168/788 (21.32%) 299/1366 (21.89%) 205/877 (23.38%)	679/808 (84.03%) 1205/1397 (86.26%) 766/896 (85.49%)
**Characteristics**	**Q6**	**Q7**		**Q8**	**Q9**
**Age**	*p* = 0.014 *	*p* = 0.067		*p* = 0.195	*p* = 0.124
	**Correct answers**	**Correct answers**		**Correct answers**	**Correct answers**
30–39 40–49 50–59 60–69	771/838 (92.00%) 797/846 (94.21%) 852/901 (94.56%) 845/884 (95.59%)	541/710 (76.20%) 560/706 (79.32%) 593/743 (79.81%) 595/727 (81.84%)		592/720 (82.22%) 612/714 (85.71%) 636/746 (85.25%) 657/767 (85.66%)	266/709 (37.52%) 282/697 (40.46%) 296/703 (42.11%) 309/711 (43.46%)
**Province**	*p* = 0.003 **	*p* = 0.533		*p* = 0.037 *	*p* = 0.215
	**Correct answers**	**Correct answers**		**Correct answers**	**Correct answers**
Alberta British Columbia Ontario Quebec	843/880 (95.80%) 791/841 (94.05%) 823/868 (94.82%) 808/880 (91.82%)	576/737 (78.15%) 549/684 (80.26%) 562/718 (78.27%) 602/747 (80.59%)		636/749 (84.91%) 598/688 (86.92%) 638/745 (85.64%) 625/765 (81.70%)	285/703 (40.54%) 301/680 (44.26%) 281/710 (39.58%) 286/727 (39.34%)
**Country of birth**	*p* = 0.403	*p* = 0.003 **		*p* < 0.001 **	*p* = 0.099
	**Correct answers**	**Correct answers**		**Correct answers**	**Correct answers**
Canada Others	2774/2941 (94.32%) 480/514 (93.39%)	1955/2434 (80.32%) 324/438 (73.97%)		2135/2489 (85.78%) 352/446 (78.92%)	956/2382 (40.13%) 190/428 (44.39%)
**Education**	*p* = 0.685	*p* = 0.679		*p* = 0.308	*p* = 0.152
	**Correct answers**	**Correct answers**		**Correct answers**	**Correct answers**
High school or less Post-secondary University degree	854/910 (93.85%) 1460/1544 (94.56%) 951/1013 (93.88%)	609/762 (79.92%) 1008/1283 (78.57%) 671/840 (79.88%)		637/747 (85.27%) 1095/1309 (83.65%) 764/889 (85.94%)	299/723 (41.36%) 491/1257 (39.06%) 363/839 (43.27%)
**Characteristics**	**Q1**	**Q2**	**Q3**	**Q4**	**Q5**
**Marital status**	*p* = 0.191	*p* = 0.387	*p* = 0.179	*p* = 0.396	*p* = 0.176
	**Correct answers**	**Correct answers**	**Correct answers**	**Correct answers**	**Correct answers**
Married or with partner Divorced, separated, or widowed Single or never married	1039/1730 (60.06%) 294/461 (63.77%) 295/507 (58.19%)	1582/2006 (78.86%) 443/576 (76.91%) 439/573 (76.61%)	833/1844 (45.17%) 248/515 (48.16%) 257/522 (49.23%)	410/1917 (21.39%) 124/537 (23.09%) 131/549 (23.86%)	1681/1958 (85.85%) 475/549 (86.52%) 469/565 (83.01%)
**Employment status**	*p* = 0.133	*p* = 0.221	*p* = 0.014 *	*p* = 0.455	*p* = 0.001 **
	**Correct answers**	**Correct answers**	**Correct answers**	**Correct answers**	**Correct answers**
Working Not working Retired	1006/1627 (61.83%) 314/533 (58.91%) 13/546 (57.33%)	1507/1915 (78.69%) 461/611 (75.45%) 498/633 (78.67%)	838/1739 (48.19%) 255/548 (46.53%) 247/598 (41.30%)	415/1815 (22.87%) 126/572 (22.03%) 127/621 (20.45%)	1573/1835 (85.72%) 482/595 (81.01%) 568/643 (88.34%)
**Family income**	*p* = 0.673	*p* = 0.006 **	*p* = 0.316	*p* = 0.894	*p* = 0.004 **
	**Correct answers**	**Correct answers**	**Correct answers**	**Correct answers**	**Correct answers**
Less than CAD 20,000 CAD 20,000–39,999 CAD 40,000–59,999 CAD 60,000–79,999 More than CAD 79,999	109/193 (56.48%) 251/414 (60.63%) 281/474 (59.28%) 238/386 (61.66%) 579/939 (61.66%)	176/234 (75.21%) 343/473 (72.52%) 431/562 (76.69%) 346/440 (78.64%) 881/1091 (80.75%)	100/218 (45.87%) 197/429 (45.92%) 219/506 (43.28%) 188/418 (44.98%) 491/1006 (48.81%)	53/215 (24.65%) 97/440 (22.05%) 122/527 (23.15%) 102/438 (23.29%) 229/1046 (21.89%)	170/221 (76.92%) 383/461 (83.08%) 454/534 (85.02%) 373/433 (86.14%) 933/1076 (86.71%)
**Insurance status**	*p* = 0.037 *	*p* = 0.001 **	*p* = 0.224	*p* = 0.387	*p* = 0.008 **
	**Correct answers**	**Correct answers**	**Correct answers**	**Correct answers**	**Correct answers**
Has insurance No insurance, interested No insurance, not interested	1119/1839 (60.85%) 286/504 (56.75%) 186/282 (65.96%)	1692/2116 (79.96%) 427/583 (73.24%) 274/366 (74.86%)	928/1995 (46.52%) 224/496 (45.16%) 167/327 (51.07%)	450/2073 (21.71%) 125/518 (24.13%) 81/339 (23.89%)	1835/2116 (86.72%) 440/525 (83.81%) 284/351 (80.91%)
**Characteristics**	**Q6**	**Q7**		**Q8**	**Q9**
**Marital status**	*p* = 0.007 **	*p* = 0.399		*p* = 0.428	*p* = 0.058
	**Correct answers**	**Correct answers**		**Correct answers**	**Correct answers**
Married or with partner Divorced, separated, or widowed Single or never married	2047/2172 (94.24%) 598/623 (95.99%) 587/639 (91.86%)	1443/1829 (78.9%) 403/494 (81.58%) 423/537 (78.77%)		1569/1846 (84.99%) 461/540 (85.37%) 445/537 (82.87%)	716/1784 (40.13%) 224/489 (45.81%) 207/524 (39.50%)
**Employment status**	*p* = 0.007 **	*p* = 0.344		*p* = 0.184	*p*= 0.047 *
	**Correct answers**	**Correct answers**		**Correct answers**	**Correct answers**
Working Not working Retired	1954/2079 (93.99%) 611/662 (92.30%) 669/695 (96.26%)	1368/1741 (78.58%) 429/541 (79.30%) 473/581 (81.41%)		1493/1778 (83.97%) 456/539 (84.60%) 526/604 (87.09%)	687/1733 (39.64%) 207/510 (40.59%) 252/553 (45.57%)
**Family income**	*p* = 0.133	*p* = 0.656		*p* = 0.527	*p* = 0.034 *
	**Correct answers**	**Correct answers**		**Correct answers**	**Correct answers**
Less than CAD 20,000 CAD 20,000–39,999 CAD 40,000–59,999 CAD 60,000–79,999 More than CAD 79,999	225/249 (90.36%) 472/503 (93.84%) 559/598 (93.48%) 452/482 (93.78%) 1137/1200 (94.75%)	164/200 (82.00%) 347/438 (79.22%) 401/497 (80.68%) 332/421 (78.86%) 779/998 (78.06%)		176/209 (84.21%) 354/426 (83.10%) 439/530 (82.83%) 365/426 (85.68%) 857/1001 (85.61%)	100/199 (50.25%) 175/420 (41.67%) 197/485 (40.62%) 176/405 (43.46%) 378/979 (38.61%)
**Insurance status**	*p* = 0.020 *	*p* = 0.566		*p* = 0.055	*p* = 0.096
	**Correct answers**	**Correct answers**		**Correct answers**	**Correct answers**
Has insurance No insurance, interested No insurance, not interested	2213/2331 (94.94%) 573/616 (93.02%) 362/394 (91.88%)	1564/1958 (79.88%) 396/498 (79.52%) 252/326 (77.30%)		1717/1999 (85.89%) 429/525 (81.71%) 279/331 (84.29%)	759/1909 (39.76%) 229/513 (44.64%) 130/300 (43.33%)

^†^ Knowledge statements excludes the “Don’t know” answer choice, *: *p* < 0.05, **: *p* < 0.01.

## Data Availability

The study questionnaire is available upon request to the corresponding author.
